# Caring for the critically ill patients over 80: a narrative review

**DOI:** 10.1186/s13613-018-0458-7

**Published:** 2018-11-26

**Authors:** Bertrand Guidet, Helene Vallet, Jacques Boddaert, Dylan W. de Lange, Alessandro Morandi, Guillaume Leblanc, Antonio Artigas, Hans Flaatten

**Affiliations:** 1 Assistance Publique - Hôpitaux de Paris (AP-HP), Service de Réanimation Médicale, Hôpital Saint-Antoine, 184 rue du Faubourg Saint-Antoine, 75012 Paris, France; 2 Sorbonne Universités, Université Pierre et Marie Curie - Paris 06, Paris, France; 3 INSERM, UMR_S 1136, Institute Pierre Louis d’Épidémiologie et de Santé Publique, 75013 Paris, France; 4 Assistance Publique - Hôpitaux de Paris (AP-HP), Service de gériatrie, Hôpital Pitié salpêtrière, 75013 Paris, France; 5 Department of Intensive Care, University Medical Center Utrecht, University Utrecht, Utrecht, The Netherlands; 6 Department of Rehabilitation Hospital Ancelle di Cremona, Cremona, Italy; 7 Geriatric Research Group, Brescia, Italy; 8 Division of Critical Care Medicine, Department of Anesthesiology and Critical Care Medicine, Université Laval, Québec City, QC Canada; 9 Centre de recherche du CHU de Québec – Université Laval, Population Health and Optimal Health Practices Research Unit (Trauma – Emergency – Critical Care Medicine), Université Laval, Québec City, QC Canada; 10 Department of Intensive Care Medecine, CIBER EnfermedadesRespiratorias, Corporacion Sanitaria Universitaria Parc Tauli, Autonomous University of Barcelona, Sabadell, Spain; 11 Department of Anaesthesia and Intensive Care, Haukeland University Hospital, Bergen, Norway; 12 Department of Clinical Medicine, University of Bergen, Bergen, Norway

## Abstract

**Background:**

There is currently no international recommendation for the admission or treatment of the critically ill older patients over 80 years of age in the intensive care unit (ICU), and there is no valid prognostic severity score that includes specific geriatric assessments.

**Main body:**

In this review, we report recent literature focusing on older critically ill patients in order to help physicians in the multiple-step decision-making process. It is unclear under what conditions older patients may benefit from ICU admission. Consequently, there is a wide variation in triage practices, treatment intensity levels, end-of-life practices, discharge practices and frequency of geriatrician’s involvement among institutions and clinicians. In this review, we discuss important steps in caring for critically ill older patients, from the triage to long-term outcome, with a focus on specific conditions in the very old patients.

**Conclusion:**

According to previous considerations, we provide an algorithm presented as a guide to aid in the decision-making process for the caring of the critically ill older patients.

**Electronic supplementary material:**

The online version of this article (10.1186/s13613-018-0458-7) contains supplementary material, which is available to authorized users.

## Introduction

In the past 20 years, there has been an increase in the elderly population admitted to the intensive care unit (ICU) [1, 2]. Currently, the median age of critically ill patients approaches 65 years in many countries, and the proportion of the very old (80 years or over) critically ill patients will increase faster than any other cohort in the ICUs [3]. Intensive care unit resources use in the very old patients carries a high burden on healthcare costs [4, 5]. In a Canadian study, the average cost of an ICU admission of patients 80 years or over was $31,679 [4]. Undeniably, caring for older patients frequently poses ethical and practical challenges both prior to and during ICU admission [6]. Such decision making requires an in-depth understanding of aging and its consequences on normal organ function, together with close communication with family and other caregivers [7].

**Fig. 1:**
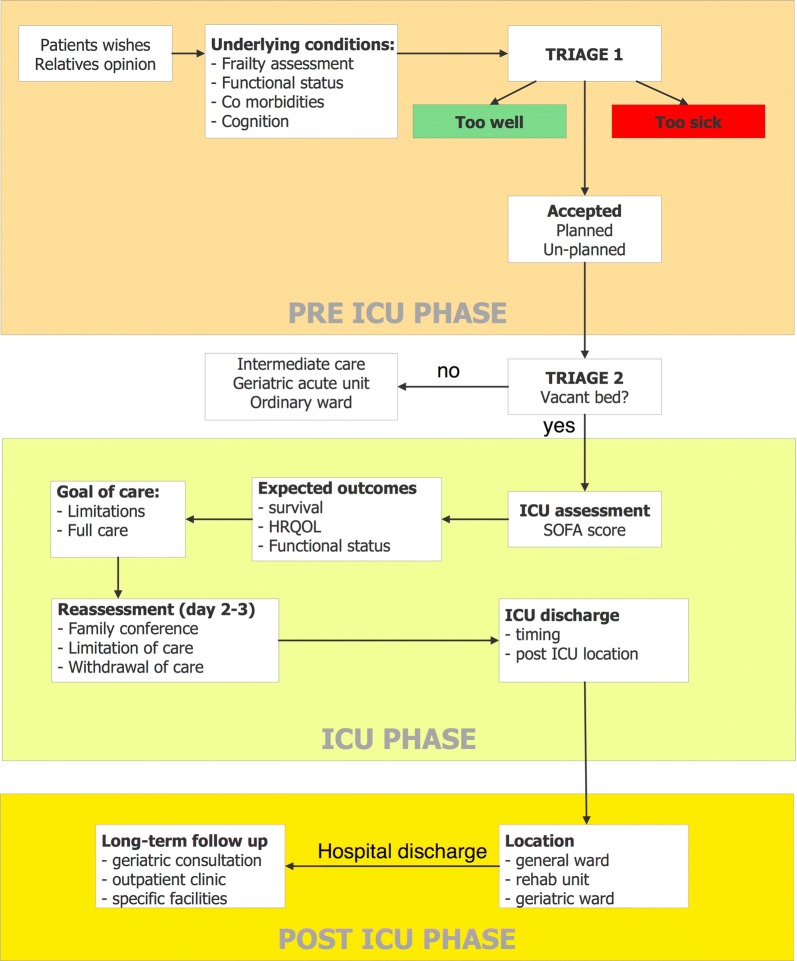
Algorithm for critically ill patients over 80y

In this review, we will discuss important steps in caring for the critically ill patients 80 years or over, from the triage to discharge, long-term rehabilitation or palliative care. We will highlight recent researches and advances in this field and propose an algorithm that can be used as a guide in the decision-making process for the caring of the critically ill older patients (Fig. 1).

## Characterization of an old patient: lessons to be learnt from geriatricians

### Aging

**Fig. 2:**
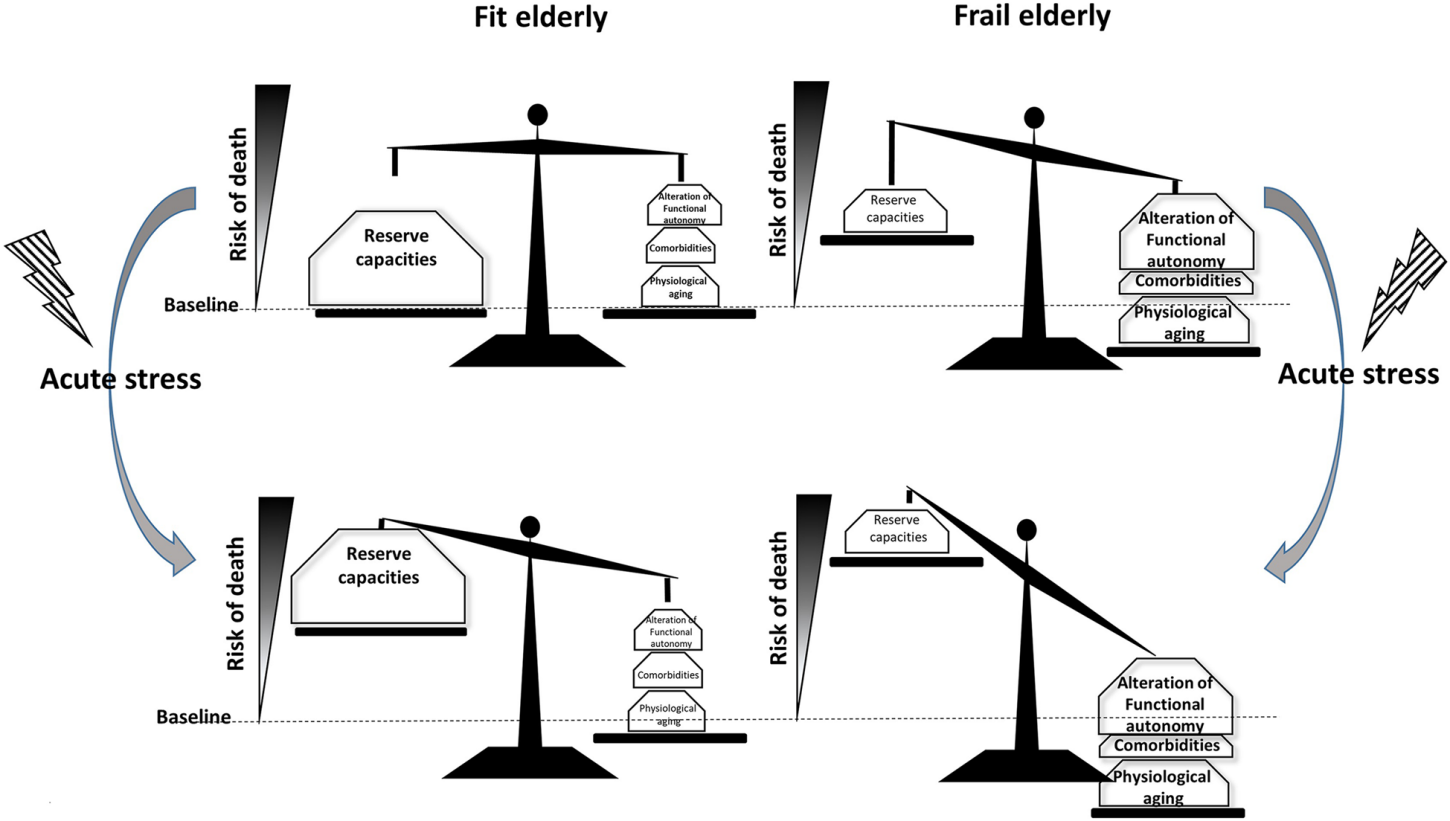
Impact of acute stress on fit or frail elderly. Physiological aging, comorbidities and functional dependency are the main components of frailty syndrome, leading to decrease in reserve capacities. At baseline, impact of frailty on survival is slight but its weight dramatically grows in case of acute stress (all medical events leading to ICU admission) and increase the risk of death comparatively to the fit elderly

There is no consensual definition of aging [8]. The World Health Organization considers anyone over 65 years old as “elderly.” However, in the ICU, we commonly characterize patients 80 years or over as “very old.” Aging is a complex transition that includes a physiological and cognitive vulnerability, making the individual more prone to diseases and acute medical events, leading to further decrease in reserve capacities, loss of functional independency and ultimately to death (Fig. 2).

### Comorbidities

The proportion of patients with comorbidities and the number of comorbidities per patients increase with age. The mean number of comorbidities per patients is 2.6 ± 2.2 in patients 65–84 years old and 3.6 ± 2.3 in patients 85 years or over [9]. The most common comorbidities are hypertension, diabetes, chronic obstructive pulmonary disease, cardiac failure, cancer and cognitive impairment [9, 10]. Comorbidities are associated with an increased mortality [11], loss in physical independency and an increase in hospitalization rates [12]. In ICUs, comorbidities are associated with higher in-hospital [13, 14] and long-term mortality rates [15]. The Charlson comorbidity index (Table 1) has been validated in critically ill patients and is predictive of mortality [15, 16].

**Table 1:** Summary of main scales used in geriatric evaluation

*Comorbidities*	
Charlson comorbidity index [16]	Age (years old)
	50–59 (1 point)
	60–69 (2 points)
	70–79 (3 points)
	≥ 80 (4 points)
	Diabetes
	Uncomplicated (1 point)
	End-organ damage (2 points)
	Liver disease
	Mild (1 point)
	Moderate to severe (3 points)
	Malignancy
	Any leukemia, lymphoma or localized solid tumor (2 points)
	Metastatic solid tumor (6 points)
	AIDS (6 points)
	Moderate-to-severe renal disease (2 points)
	Congestive heart failure (1 point)
	Myocardial infarction (1 point)
	Chronic pulmonary disease (1 point)
	Peripheral vascular disease (1 point)
	Cerebrovascular disease (1 point)
	Dementia (1 point)
	Hemiplegia (2 points)
	Connective tissue disease (1 point)
	Peptic ulcer disease (1 point)
*Functional autonomy*	
ADL scale [30]	Bathing (independent: 1 point; partially dependent: 0,5 point; totally dependent: 0 point)
	Dressing (independent: 1 point; partially dependent: 0,5 point; totally dependent: 0 point)
	Toileting (independent: 1 point; partially dependent: 0,5 point; totally dependent: 0 point)
	Transfer (independent: 1 point; partially dependent: 0,5 point; totally dependent: 0 point)
	Continence (independent: 1 point; partially dependent: 0,5 point; totally dependent: 0 point)
	Feeding (independent: 1 point; partially dependent: 0,5 point; totally dependent: 0 point)
Scale from 0 (totally dependent) to 6 (independent)	
IADL scale [31]	Ability to use telephone
	Operates telephone on own initiative; looks up and dials numbers (1 point)
	Dials a few well-known numbers (1 point)
	Answers telephone, but does not dial (1 point)
	Does not use telephone at all (0 point)
	Shopping
	Takes care of all shopping needs independently (1 point)
	Shops independently for small purchases (0 point)
	Needs to be accompanied on any shopping trip (0 point)
	Completely unable to shop (0 point)
	Food preparation
	Plans, prepares and serves adequate meals independently (1 point)
	Prepares adequate meals if supplied with ingredients (0 point)
	Heats and serves prepared meals or prepares meals but does not maintain adequate diet (0 point)
	Needs to have meals prepared and served (0 point)
	Housekeeping
	Maintains house alone with occasion assistance (heavy work) (1 point)
	Performs light daily tasks such as dishwashing, bed making (1 point)
	Performs light daily tasks, but cannot maintain acceptable level of cleanliness (1 point)
	Needs help with all home maintenance tasks (1 point)
	Does not participate in any housekeeping tasks (0 point)
	Laundry
	Does personal laundry completely (1 point)
	Launders small items, rinses socks, stockings, etc. (1 point)
	All laundry must be done by others (0 point)
	Mode of transportation
	Travels independently on public transportation or drives own car (1 point)
	Arranges own travel via taxi, but does not otherwise use public transportation (1 point)
	Travels on public transportation when assisted or accompanied by another (1 point)
	Travel limited to taxi or automobile with assistance of another (0 point)
	Does not travel at all (0 point)
	Responsibility for own medications
	Is responsible for taking medication in correct dosages at correct time (1 point)
	Takes responsibility if medication is prepared in advance in separate dosages (0 point)
	Is not capable of dispensing own medication (0 point)
	Ability to handle finances
	Manages financial matters independently (budgets, writes checks, pays rent and bills, goes to bank); collects and keeps track of income (1 point)
	Manages day-to-day purchases, but needs help with banking, major purchases, etc. (1 point)
	Incapable of handling money (0 point)
Scale from 0 (low function/dependent) to 8 (high function/independent)	
*Frailty*	
Rockwood Clinical Frailty Scale [36]	1. Very fit—People who are robust, active, energetic and motivated. These people commonly exercise regularly. They are among the fittest for their age
	2. Well—People who have no active disease symptoms but are less fit than category 1. Often, they exercise or are very active occasionally, e.g., seasonally
	3. Managing well—People whose medical problems are well controlled, but are not regularly active beyond routine walking
	4. Vulnerable—While not dependent on others for daily help, often symptoms limit activities. A common complaint is being “slowed up,” and/or being tired during the day
	5. Mildly frail—These people often have more evident slowing and need help in high-order IADLs (finances, transportation, heavy housework, medications). Typically, mild frailty progressively impairs shopping and walking outside alone, meal preparation and housework
	6. Moderately frail—People need help with all outside activities and with keeping house. Inside, they often have problems with stairs and need help with bathing and might need minimal assistance (cuing, standby) with dressing
	7. Severely frail—Completely dependent for personal care, from whatever cause (physical or cognitive). Even so, they seem stable and not at high risk of dying (within ~ 6 months)
	8. Very severely frail—Completely dependent, approaching the end of life. Typically, they could not recover even from a minor illness
	9. Terminally ill—Approaching the end of life. This category applies to people with a life expectancy

*AIDS* Acquired Immunodeficiency Syndrome, *ADL* activity of daily living, *IADL* Instrumental Activities of Daily Living

Physiological changes leading to the decrease in reserve capacities have been extensively described [17–20]. Advanced age leads to an alteration in respiratory physiology (loss of elastic lung tissue, increased anteroposterior diameter of the chest, decreased muscle strength and sensitivity of respiratory centers to hypoxemia and hypercapnia) leading to increased risk of acute respiratory failure and mortality. The combination of immunosuppression and “inflamm-aging,” called “immunosenescence,” results in higher rates of viral reactivation and infection susceptibility and severity.

### Malnutrition

Malnutrition affects 12–45% of hospitalized older patients and is associated with longer hospital length of stay, poor physical independency, poor quality of life and higher mortality [21, 22]. Furthermore, malnutrition importantly contributes to “frailty” [23]. In critically ill patients, malnutrition and negative protein–energy balance are associated with higher ICU length of stay, mortality, rate of acquired infection and length of mechanical ventilation [24].

### Cognitive impairment

Prevalence of preexisting cognitive impairment at ICU admission ranges from 6 to 43% [25, 26]. In the ICU, cognitive impairment is a strong risk factor for delirium [27, 28], which is associated with increased mortality and with subsequent further cognitive and executive function decline [25, 29].

### Functional decline and frailty

General assessment of baseline functional status is crucial. Several scales estimate physical dependency, like the Activities of Daily Living scale and Instrumental Activities of Daily Living scale (Table 1) [30, 31]. In clinical practice, a simplified version of Instrumental Activities of Daily Living scale is used [32].

The “frailty syndrome” is defined as an individual’s inherent vulnerability leading to difficulties to overcome acute stress [33, 34]. The frailty model includes areas such as physiological functioning, comorbidities, functional impairment and social difficulties [34, 35]. An easy-to-use Clinical Frailty Scale (CFS) was proposed in 2008, ranking patients in nine group from “very fit” to “terminally ill” (Table 1) (Additional file 1) [36, 37]. An increased CFS is associated with a higher risk of mortality [36]. Furthermore, frailty is associated with a higher risk of falls, worsening mobility and institutional care [38]. A recent meta-analysis concludes that there is a higher risk of not returning to home and a higher risk of in-hospital and long-term mortality in frail patients admitted to the ICU [33]. In the VIP1 study, which included 5132 critically ill patients ≥ 80 years from 311 European ICUs, frailty (CFS > 4) was present in 43.1% and was independently related to ICU (22.2%) and 30-day mortality (35.8%) [39].

## Medications: a special concern in old patients

The problem of medication and its biodisponibility in old patients have been well described since 1980 [40, 41]. Polypharmacy and inappropriate medications prescription among older patients are receiving increasing attention [42, 43] as it frequently leads to adverse outcomes [44, 45]. In particular, acute hospitalizations pose an increased risk of inappropriate prescription because of newly prescribed medications, the presence of multiple prescribers, inadequate medication reconciliation and a lack of care coordination [46].

Medications typically intended for short-term use during acute illness are sometimes continued after discharge without a documented indication [47]. While it is possible that these drugs were appropriately started during an acute illness in the ICU, most should have been discontinued at ICU or hospital discharge [48]. A common complication of critical illness is an increase in psychological symptoms, sleep cycle alterations, delirium and cognitive impairment, which is associated with increased prescription of specific medications, such as antipsychotics or benzodiazepines [45, 49, 50]. Despite the lack of reliable evidence supporting their use in the ICU, antipsychotics are routinely used in critically ill patients [51]. One potential drawback of antipsychotic use in the ICU is their continuation after the transition to other clinical settings, including discharge from the hospital [52]. Between 12 and 32% of older ICU survivors are discharged with an antipsychotic despite the fact that the majority of these patients were no longer delirious [48, 52–54]. Antipsychotics may even increase the risk of long-term mortality, especially in patients with dementia [55]. The use of these drugs has strongly been discouraged by the American Geriatrics Society and National Institute for Clinical Excellence because of their potential harmful effects [56].

Benzodiazepines and sedative hypnotics are commonly used to treat insomnia and agitation in older adults despite significant risks. Benzodiazepine administration was found to be an independent risk factor for delirium [50, 57]. Clinicians should use alternatives known to reduce the daily number of benzodiazepines such as use of dexmedetomidine or propofol [58, 59].

Early detection of inappropriate medication prescriptions may prevent adverse drug events and improve geriatric care [60, 61]. Different criteria are available to support a multidisciplinary team in medications evaluation such as the Beers Criteria for Potentially Inappropriate Medications Use in Older Adults [62]; the STOPP (Screening Tool of Older Persons’ potentially inappropriate Prescriptions) and START (Screening Tool to Alert doctors to the Right Treatment) criteria [63].

## Triage: a multidisciplinary approach including the patient’s wishes

Intensive care unit triage is triggered by a formal or informal contact from a referring physician to a consultant physician. Both physicians, together with the patient and relatives, should consider the potential benefit from an ICU admission. If there is potential benefit considered, the patient should be proposed for admission to the ICU. If the benefit of an ICU admission is considered to be low, other options may be considered, from admission to a high-dependency unit or intermediate care to treatment on a regular or palliative care ward. It should be emphasized that there is often a mismatch between the clinicians’ assessment and the patient’s wishes [64]. Multidisciplinary collaboration in the decision-making process is strongly advocated [65]. More specifically for the very old, all healthcare providers who can help to improve the decision-making process for the benefit of the patient should be involved. Several important aspects should be considered by clinicians in the decision-making process: self-reflective and empowering leadership by physicians; practice and culture of open interdisciplinary reflection; culture of not avoiding end-of-life decisions; culture of mutual respect within the interdisciplinary team; active involvement of nurses in end-of-life care and decision making; active decision making by physicians; and practice and culture of ethical awareness.

Many patients are triaged by the emergency physician or the treating physician on the ward before the ICU consultation. This “hidden triage” is often not captured by studies focusing only on patients proposed for ICU admission [66]. However, even when proposed for ICU admission many old patients are declined. In a single-center study, 73.3% (132/180) of older patients referred for ICU admission were declined [67]. Similar data were recently found in a Norwegian multicenter study [68]. In the ICE-CUB 1 study [66], the ICU admission rate in critically ill older patients was only 14.4% after being triaged by the emergency physician and the intensivist. It is remarkable to notice that in these three studies, the long-term mortality was inferior to 100% in patients considered too sick for an ICU admission. On the other hand, the mortality was far from 0% in patients considered too well for an ICU admission (Table 2). These results suggest over- and under-utilization of ICUs. However, in the large multicenter observational Eldicus study (*n* = 6796 patients), 82% of all patients were admitted, while nearly half of the patients (49%) were ≥ 65 years. Although a higher proportion of older patients were refused ICU admission, the survival benefit of admitted versus non-admitted patient seemed to increase with age [69].

**Table 2:** Results from three studies of pre ICU triage in very old patients

Patients triaged	Hospital mortality (%)	Long-term mortality (%)
Garrouste-Ortegas [67]		At 1 year
Admission (*n* = 48)	62.5	70.8
Too sick (*n* = 79)	70.8	87.3
Too well (*n* = 51)	17.6	47
Boumendil [109]		At 6 months
Admission (*n* = 316)	32.7	47.5
Too sick (*n* = 821)	58	81.1
Too well (1339)	10.1	33.1
Andersen [68]		At 1 year
Admission (*n* = 250)	44	60
Too sick (*n* = 52)	67.3	88.5
Too well (*n* = 46)	34.8	50

### Advanced directives should be available for older patients

When an ICU admission is considered, clinicians must ensure that invasive procedures consistent with intensive care are in accordance with the patient’s wishes. Patient physical dependency and previous documented decisions about end of life must be respected. However, very often realistic advanced healthcare directives have not been discussed before ICU admission. Whenever this is the situation, there are three possibilities:

- The patient has a normal cognition and is able to consent to care. Usually such conversation should be undertaken in the presence of the family or caregivers.
- Frequently, the old patients are unable to consent to care. In that case, physicians must discuss the intensity of care with the surrogate decision makers (family or caregivers). The main question is not what the surrogate decision maker think about and ICU admission, but what they know about the patient’s wishes and how the patient would have responded to being admitted to intensive care.
- In emergency situations, there is no time for information retrieval from the surrogate decision makers and treatments are usually started without informed consent. In most countries, it is then possible to withdraw life-sustaining therapies (LSTs) when more information is available [70] (see small case story in Additional file 2).

## Level of treatment during the intensive care unit stay

When an older patient has been admitted to the ICU, the most appropriate treatment should be given. However, this does not necessarily mean maximal treatment. If, during the shared decision-making process, certain treatments such as invasive mechanical ventilation are thought to be disproportional to the chances of survival or certain treatments are refused by the patient, these treatments should not be imposed upon the patient [64]. However, to give a patient a fair chance all other treatments should be applied. The ethical climate has also been found to have an impact on treatment-limitation decisions and time until death [71].

### Severity of the disease

The severity scores have a poor discrimination in old patients [3] because they do not consider any geriatric assessment. A Canadian study developed a prediction tool for futility of ICU care although this study experienced extremely high observed mortality in the higher percentiles of risk [72].

### Withholding and withdrawing treatment

Older patients often receive a lower level of treatment intensity than their younger counterparts. For example, the prevalence of limitations of life-sustaining therapies increased with age in surgical population [73–75]. In addition, decisions to withhold LST were made earlier during the ICU stay in comparison with younger patients [13, 76]. In patients without improvement of their clinical situation, the therapeutic intensity level may no longer be in accordance with the patients’ chances of long-term survival with acceptable quality of life, and a clinical decision might need to be made. Obviously, the timing of such a decision is arbitrary, but most agree that the older patient should be offered an “ICU trial” that lasts long enough to observe possible improvements [77]. Life-sustaining therapies limitation is not equivalent to end-of-life decision. In the VIP1 study including more than 5000 patients ≥ 80 years, the ICU and 30-day mortality rates were, respectively, 29% and 53% in the withholding group, and 82% and 93% in the withdrawing group [70]. In another study, ICU and hospital mortality rates were 56% and 69% despite a decision to withhold or withdraw LST [78].

For patients with withholding or withdrawing of LST, an important goal is to achieve the most comfortable death [78]. Family members reported that the “patient be comfortable and suffer as little as possible” was their most important value and “the belief that life should be preserved at all costs” was their least important value considered in making treatment decisions [79]. Mobile palliative care team could be very useful to help in the decision process and even to propose admission in a palliative unit.

In a recent study focusing on patients older than 80, LST limitation was common (27.2%) but with important regional differences [70]. In the Northern region, 45.2% had LST withdrawn compared with 12.8% in the Eastern region.

Apart from patient-related factors, other reasons to limit LSTs might play a role. Apparently, the ICU bed availability is associated with the timing of limitations of LSTs. Patients admitted in ICUs with a lower bed availability had a shorter time to do-not-resuscitate decisions and patients who had do-not-resuscitate decisions had shorter time to death [80]. In the VIP1 study, there was no relation between number of ICU beds and percentage of LST limitations. Percentage of LST limitations was higher in countries with high growth domestic product and was lower in more religious countries [70].

## Situations with specificities related to age

*Acute respiratory failure* (ARF) was the most frequent reason for urgent ICU admission in the VIP1 study (25%). Mechanical ventilation is responsible for a large percentage of ICU costs [81]. Experimental studies suggest that aging is also associated with an increased susceptibility to ventilator-induced lung injury [82]. The high mortality could be related to delays in diagnosis and treatment [83] since the presenting signs and symptoms may not be primarily respiratory such as delirium and cognitive impairment.

The incidence of acute respiratory distress syndrome (ARDS) increases markedly with age, which is largely determined by a higher incidence of sepsis in the very old [84]. Several studies have shown that age is independently associated with mortality in patients requiring mechanical ventilation and in ARDS [85, 86]. Age was also associated with longer duration of mechanical ventilation, ICU length of stay and mortality in ARDSNet database [87]. Long-term sequelae have been described after ARDS and are more frequent and severe in older patients. The ability to sustain spontaneous respiration after extubation is lower in patients older than 70 years, mainly due to the inability to clear tracheobronchial secretions and a higher incidence of nosocomial pneumonia [87].

*Sepsis* The incidence of sepsis increases with age. Additional risks include: subtle clinical presentations, institutionalization, use of invasive devices, multiple medications, reduced renal function and poor nutritional status. In the elderly population, sepsis is a major cause of morbidity and mortality, with almost 60% of septic patients being over 65 years of age [88].

Typically, younger patients with sepsis demonstrated fever, tachycardia and capillary vasodilatation. Older patients often exhibit few of these clinical symptoms but may present with an altered mental status, making early recognition rather challenging. Elderly patients often present with heart failure, arrhythmias and arterial hypertension. They have a lower cardiac compliance, whereas the renal perfusion is more cardiac flow dependent; thus, the management of fluid therapy is more sensitive. A predefined volume administration (30 mL/kg) is inappropriate in most patients. Fluid bolus (250 ml at 5- to 10-min intervals) and further evaluation to test whether goals are reached is a reasonable option.

*Scheduled surgery should be considered apart from other stays* Postoperative patients after planned surgery have a much better long-term survival than acute or emergency admissions [39, 89]. Obviously, patients after elective surgery represent a highly selected population of older patients. These patients tend to be younger, less frail and with a lower SOFA score on the first day of admission than patients after emergency admission [39]. In the VIP1 study, postoperative elective ICU admissions (*n* = 906) accounted for 17.7% of all admission and 24.6% of ICU stays shorter than 24 h. Most of the patients were not frail, and the 1-month mortality was much lower than for acutely admitted patients [39]. In a study by Bagshaw, 72% of critically ill patients over 80 years admitted to the ICU were discharged home after elective surgery [13]. Long-term prognostic is much better for scheduled surgery compared to urgent admission [90].

No validated score is currently available to predict the need of perioperative ICU admission in the older patients. In a study of 275 patients aged 65 years or over undergoing intermediate- or high-risk elective surgery, a multidimensional frailty model (composed of the Charlson comorbidity index, dependence in activities of daily living, dementia, risk of delirium, short mid-arm circumference and malnutrition) predicted mortality rates more accurately than the ASA classification (84% sensitivity and 69% specificity). High-risk patients (defined as a multidimensional frailty score > 5) showed increased postoperative mortality risk and longer hospital LOS [91]. In contrast, the urgent surgical patients requiring ICU admission are as severely ill as medical patients need more organ support and have longer hospital LOS (see Additional file 3).

## Timing and location of ICU discharge are keys elements for the outcome

Risk factors for in-hospital mortality after ICU discharge include age, comorbidities and severity of illness [92], in addition to organizational factors, such as discharge time and the availability of step-down facilities [93, 94]. Older patients discharged from the ICU are particularly vulnerable to poor handovers due to their complicated physiology and the substantial decrease in monitoring when these patients are transferred from the ICU to a general ward [95, 96]. However, studies have failed to show an impact of discharge protocol on hospital mortality [96], although none tested the discharge location as a potential variable of interest [95]. Except for acute geriatric units (AGUs), geriatric expertise is usually not available on a regular basis in other wards. To illustrate this, of the participants of the VIP1 study [39], one-third disagreed that a consultation of a geriatrician should be sought when deciding to discharge an elderly patient [97]. Yet, due to their expertise in the field of multimorbidities and acute stress in older patients, geriatricians make a more comprehensive assessment of old patients that may lead to better care and orientation decisions in these patients. Including a geriatrician in shared decision making for old critically ill older patients may improve their outcome. However, no large-scale study supports this hypothesis.

In a cohort of 1283 French patients of ≥ 75 years, 40.3% of the patients were transferred to a geriatric unit after an emergency department visit [98]. Four factors were related to admission to a geriatrics unit: cognitive disorder, “failure to thrive” syndrome, depression or loss of Activities of Daily Living. However, considerable nation-wide variations were observed underlying the need to clarify and reinforce this discipline in the emergency healthcare system. A small-scale, single-center study illustrates the beneficial effect of geriatric intervention on the functional dependency of 45 older patients after discharge from a medical/surgical ICU [99]. Although physical function was usually recovered rapidly, the degree of recovery depended on the patient’s previous physical dependency.

Inclusion of geriatric consultations has proven valuable in other areas of medicine. Studies have documented that for postoperative older patients, mainly after hip fracture, geriatric unit admission offers a benefit as compared to surgical unit admission [100]. Postoperative admission to a dedicated geriatric unit reduced both re-admission rate and 6-month mortality. After adjustment for comorbidities, risk ratio of death at 6 months was of 0.43 (95% CI 0.25–0.73, *P* = 0.002) [101].

## Long-term outcomes are the best criteria to judge appropriateness of decision (admission, LST during the ICU stay)

Predicting long-term survival and quality of life is difficult [102]. The outcome of an older patient admitted to the ICU is dependent on: previous comorbidities, diagnosis at admission, severity of the acute illness at the time, course of the disease during the ICU stay, limitations of LSTs and age itself. There is a confounding factor in the data reporting the long-term survival and quality of life, since patients might have died of limitation of LST.

The quality of life and recovery has been shown to be poorer in elderly patients ventilated for more than 7 days, with higher proportion of debilities [103]. Similar finding were reported for neurocognitive problems such as PTSD or cognitive impairment [25].

**Fig. 3:**
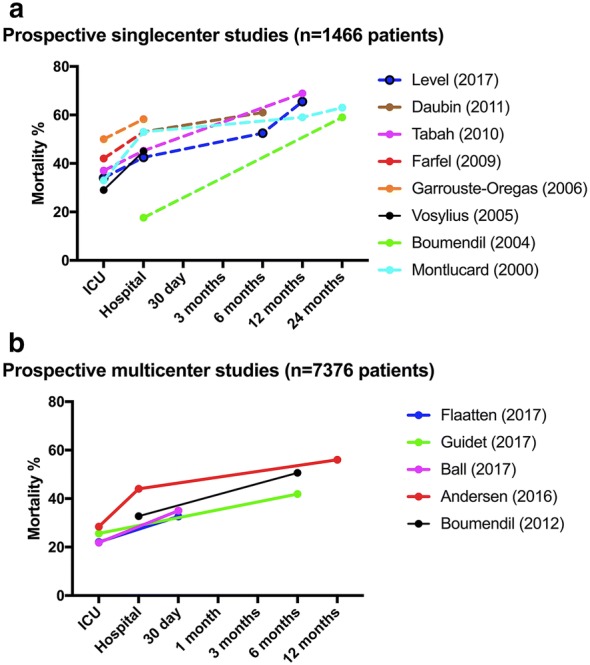
Mortality. **a** Represents mortality rates in critically ill elderly patients following admission to the ICU at ICU and hospital discharge, at 30-day and 3, 6, 12 and 24 months in single-center studies from 2000 to 2017. **b** Represents mortality rates in critically ill elderly patients following admission to the ICU at ICU and hospital discharge, at 30-day and 3, 6, 12 and 24 months in multicenter studies from 2012 to 2017

A higher proportion of older patients will be expected to wish limitation of care when having a critical illness. Outcome in terms of survival (Fig. 3), physical function at 1 year [104] and quality of life [105] in elderly ICU survivors are often reported to be significantly lower than in their younger counterparts (Table 3).

**Table 3:** Long-term functional outcome

References	Tool	Design	Age-group	Patient followed	Main results	Comparison with baseline data (ICU admission)
Kass [123]	ADL	Prospective and retrospective	> 80 years	38/105 (36.1%)	Nonsignificant decline of ADL score at 1 year	Yes
Chelluri [124]	ADL	Prospective	> 75 years, 65–74 years)	96 18/54 (33%) 20/43 (46.5%)	No difference between two age-groups at 1, 6, 12 months	Yes Method?
Broslawski [125]	ADL, IADL, GDS	Prospective	> 70 years	27/45 (60%)	Changes at 6 months related to ICU LOS and severity but not to age	Yes Method?
Montuclard [126]	ADL	Retrospective	> 70 years with 30 days of MV	30/75 (40%)	Decrease in all domains except feeding at 6 months	Retrospective estimation by the patient
Udekwu [127]	ADL	Retrospective	> 70 years	342/672 (50.8%)	At 21 months, significant decrease in ADL with more dependent patients	Yes Method?
Garrouste-Orgeas [37]	ADL	Prospective	≥ 80 years	9/48 (18%)	No change	Retrospective estimation by the patient
Kaarlola [119]	EQ-5D SF-36	Retrospective (in survivors)	65–69 years 70–74 years 75–79 years 80–94 years	114 117 91 50	More than 50% assessed their overall health status as satisfactory. Largest % in those ≥ 80	No
Tabah [128]	ADL	Prospective	≥ 80 years	23/106 (21%)	No change 74% of patients were fully independent	Prospective estimation by the patient or relatives
Boumendil [109]	ADL	Prospective	≥ 80 years	162/329	At 6 months 16.2% were unable to perform at least one activity that they had been able to perform at the time of the ED visit	Prospective estimation by the patient or relatives
Andersen [129]	EQ-5D	Retrospective	≥ 80	58/395	HRQOL comparable with a comparison group (1 year)	
Andersen 2017 [68]	EQ-5D	Prospective	≥ 80	62/250	Lower HRQOL than a comparison group (1 year)	Compared with a age and gender reference population *n* = 179
Heyland [104]	SF-36 (physical function)	Prospective	≥ 80	505/610	50% dead and 26% achieved physical recovery at 12 months	PF compared with baseline values at admission
Level 2017 [130]	ADL, Barthel index	Prospective	≥ 75	65/188	83% of 1-year survivors lived in their own home	ADL compared with baseline at admission
Guidet [110]	ADL	Prospective	≥ 75 years	1528/3036	Selection criteria: preserved baseline ADL (median 6) At 6 months, decrease in ADL of 0.5 points	Prospective estimation by the patient or relatives

Older patients surviving the ICU often suffer from sequels, including increased long-term mortality [89], poor quality-adjusted survival [107], cognitive impairment and functional disability [25, 106–108]. Many of these events occur beyond the scope of intensive care. However, it is of outmost importance to try to predict the functional outcome (Table 3) of this very elderly patient group [102] as their focus is less on “longevity” but more on “quality of life.” The very old ICU patient is at risk of complications resulting from heavy sedation, prolonged ventilation, immobilization, insufficient nutrition, etc. Ultimately this leads to functional decline. In a prospective study, including 2646 older patients, among the survivors after 6 months only one-third was independent for all activities listed in Katz’s scale, while 16.2% were unable to perform at least one activity that they had been able to perform at the time of the ED visit [109]. The pessimistic conclusion was that, at 6 months after the ED visit, 63% of patients had either died or experienced functional deterioration. This is corroborated by a recent Canadian study in older patients with ICU LOS of more than 24 h. The survivors reported significantly worse physical functioning after 3, 6 and 12 months compared with age- and gender-matched controls [104]. In the ICE-CUB 2 study, including 3036 patients (mean age 85 years), the ADL scale decreased in at least one domain in 64% of the patients at 6 months [110]. However, other studies find that only 28–37% did not restore their previous functional dependency, evaluated on activity of daily living at 3, 6 and 12 months [111, 112]. At 12 months, 50% of survival patients recovered their previous ADL, IALD and physical capacities [104, 113]. Furthermore, 72–77% of patients return at home after ICU [13, 112, 114]. It is interesting to point at the lack of data concerning comparison of functional dependency recovery between “young” and “old” patients, while almost 25% of “young” patients do not restore their functional dependency after ICU [115].

A decline in functional performance is accompanied by a decrease in health-related quality of life (HRQoL) as demonstrated in the ICE-CUB 2 study [110]. In an UK study [116], the physical and the mental component of HRQoL short-form questionnaire (SF36) did not improve from 6 to 12 months after ICU discharge. In a Scandinavian study, the HRQoL was lower for the very elderly than for younger patients, although 97% of the elderly survivors lived at home and 88% of them considered their QoL satisfactory or good after hospital discharge [117]. Indeed, other studies show that the long-term HRQoL appears to be similar to age-matched populations [67, 112, 114, 118]. Ultimately, in the worse scenario the patients are at risk of loss of function, inability to return home, requirement for a nursing home and/or remain bed bound for the rest of their lives.

### Quality-adjusted life years

Kaarlola et al. [117] showed that the QALYs derived from ICU admission of patients 80 years or over would be a median of 4.1 years, in the 65- to 79-year-old group it would be 10.2 years and in patients less than 65 years it would be 22 years.

### House caregivers

One of the most neglected groups is the caregivers of the very old ICU survivors. After hospital discharge, many patients still need help in many activities of daily living, often provided by family members or partners. This radically changes their role from “loved one” to “caregiver.” However, many of the family members of surviving older critically ill patients are old themselves. As such, they can be considered as “the second victim” and may suffer from the same cognitive and even functional decline as the ICU survivors. For example, in a general ICU population 67% of the caregivers reported depressive symptoms, which remained in 43% at a follow-up of 1 year [119]. Variables that were significantly associated with worse mental health outcomes in caregivers were greater effect of patient care on other activities, less social support, and therefore less sense of control over life. Post-traumatic stress disorder (PTSD) and strain are seen in 21% of the caregivers [120]. A randomized trial, where caregivers provided respiratory physiotherapy at home, showed improvement of the cardiorespiratory status of the patients and their HRQoL (as measured with the EuroQoL 5D methodology) [121, 122]. While the majority of the above-mentioned studies were not specifically designed for the very elderly patient group, it seems logical that the perceived strain, depressive symptoms and HRQoL will also translate to this group of caregivers.

## Conclusions and algorithm

In this review, we have given an up-to-date review of the present knowledge about caring for the older ICU patients. This specific group will in the future certainly claim their rights to receive high-quality health care including intensive care. The outcome of the very old has improved over the past decades but remains poorer than for younger patients. Several factors account for this high mortality and more related to underlying disease than age by itself. Advanced care planning will become more and more important in the future because of the increasing number of admission in combination with technological innovation. We summarized in a table, the keys elements that should be considered when deciding to admit a patient older than 80 in ICU (Table 4). There are still many unresolved questions [3] (Table 4), and it is important to get answers to these unresolved issues before the huge « age-tsunami » reaches the hospitals within 10–15 years.

**Table 4:** Key messages and *unresolved issues*

*Triage*	
Seek for advance directives—*How promoting diffusion?*	
Every time it is possible, ask the patient about his/her wishes	
If the patient is unable to communicate, seek for relatives/family wishes	
Try to estimate the immediate and long-term risk of death considering	
Patient baseline characteristics:	
Age	
Functional status (Clinical Frailty Scale, frailty phenotype, Performance status)	
Comorbidities including cancer	
Nutritional status and protein–energy balance	
Cognitive and psychiatric disorders	
Type of admission: scheduled versus urgent	
Reason for admission	
Acute severity—*a specific score tailored to old patient should be available*	
Mobilize geriatric expertise if possible—*impact should be proved by interventional studies*	
Define a goal of care anticipating second evaluation after few ICU days—*Impact on triage, mortality, LOS, LST limitation?*	
If the patient is denied ICU admission consider palliative care	
*During the ICU stay*	
Organ support guidelines might not be appropriate for old patients—*Interventional studies focusing on older adults*	
Fluid loading	
Ventilator settings	
Weaning strategy	
Special attention to medication with high risk of	
Overdose	
Interaction	
Consider LST limitation in case of poor response to initial treatment—*Harmonize practice within and between countries*	
ICU discharge—*Intervention that should be tested in prospective trials*	
Patients are seen by a geriatrician after ICU discharge	
They are discharged to specialized geriatric unit	
Discuss timing	
*Long-term outcomes*	
*Test the impact of early rehabilitation on mortality, HRQOL and functional status*	
Consider the burden for the house caregivers	

## Additional files

**Additional file 1.** Clinical Frailty Scale (with permission).

**Additional file 2.** Case vignette.

**Additional file 3.** Outcomes.

## Abbreviations

ICU: Intensive care unit
ADL: Activities of Daily Living
IADL: Instrumental Activities of Daily Living
CFS: Clinical Frailty Scale
ICE-CUB: Intensive Care for Elderly-CUB Rea Network
APACHE: Acute Physiology and Chronic Health Evaluation
SAPS: Simplified Acute Physiology Score
SOFA: Sequential Organ Failure Assessment
LST: life-sustaining treatments
ARF: acute respiratory failure
ARDS: acute respiratory distress syndrome
STOPP: Screening Tool of Older Persons’ Potentially Inappropriate Prescriptions
START: Screening Tool to Alert doctors to the Right Treatment
VIP: Very elderly intensive care patient
ED: emergency department
HRQoL: health-related quality of life
QALYs: quality-adjusted life years
PTSD: post-traumatic stress disorder
