# Supplementary material for: Caring for the critically ill patients over 80: a narrative review
Source: Ann Intensive Care. 2018 Nov 26;8:114. doi: 10.1186/s13613-018-0458-7 (PMC6261095; doi:10.1186/s13613-018-0458-7)
Supplement: Supplementary file 1 — Additional file 1. Clinical Frailty Scale (with permission). [file 13613_2018_458_MOESM1_ESM.doc]

Electronic Supplementary Material

### Additional file 1. Clinical Frailty Scale


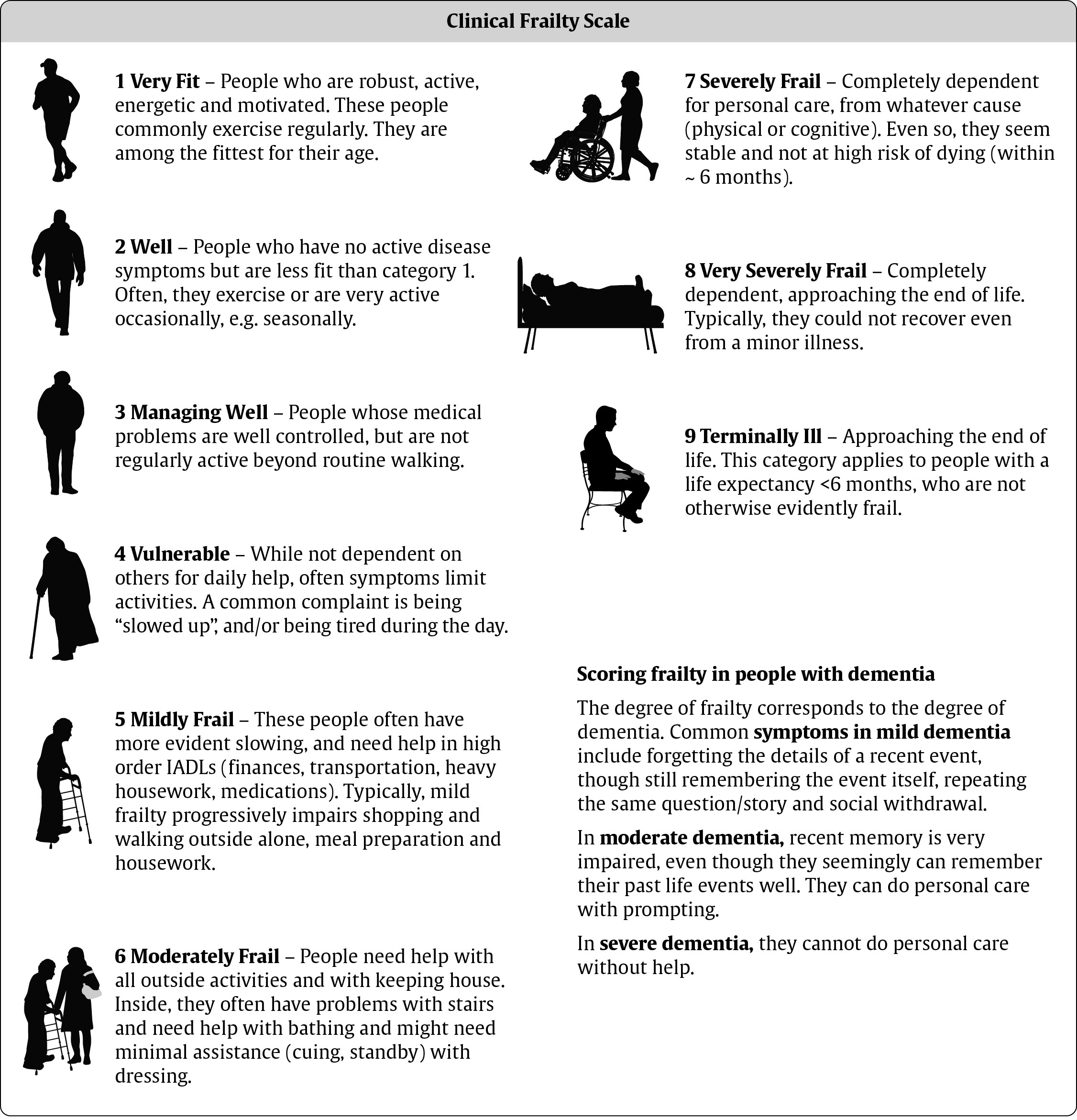


Permission to use this scale was granted from Dalhouse University, Ca, May 15 2017
