# Supplementary material for: Caring for the critically ill patients over 80: a narrative review
Source: Ann Intensive Care. 2018 Nov 26;8:114. doi: 10.1186/s13613-018-0458-7 (PMC6261095; doi:10.1186/s13613-018-0458-7)
Supplement: Supplementary file 2 — Additional file 2. Case vignette. [file 13613_2018_458_MOESM2_ESM.doc]

### Additional file 2. case vignette

An 87 years old male have been admitted to a surgical ward because of abdominal pain and constipation. He is at present under active investigation. You are called to see him because of an unwitnessed cardiac arrest at the ward, and the CA team is on its way. It is not time to consult his wife or other family members. There are no advanced directives or information about his wishes to be found in the hospital files. He is hence resuscitated and receive ROSC after 25 minutes. Now, he is unconscious and with insufficient ventilation, and you prepare to take him to the ICU. When you arrive there, his son has already been informed, and arrives at the same time. He can inform you that his father would not wish to be kept artificially alive if his heart stopped. At the same time a resident arrives and can inform you that a CT performed the same afternoon shows a large tumour in the sigmoid colon, with multiple metastatic to the liver and lungs. Further ventilation is stopped, and the patient dies after 10 minutes.
