# Supplementary material for: Caring for the critically ill patients over 80: a narrative review
Source: Ann Intensive Care. 2018 Nov 26;8:114. doi: 10.1186/s13613-018-0458-7 (PMC6261095; doi:10.1186/s13613-018-0458-7)
Supplement: Supplementary file 3 — Additional file 3. Outcomes. [file 13613_2018_458_MOESM3_ESM.doc]

Additional file 3. ICU stays characteristics of medical and surgical patients

(CUB-REA data 2016)
